# Anthelmintic Effect of Cannabidiol against *Echinococcus granulosus sensu stricto*

**DOI:** 10.3390/tropicalmed9020035

**Published:** 2024-01-31

**Authors:** Clara María Albani, Giselle Fuentes, Cristina Lujan Ramírez, Patricia Eugenia Pensel, Florencia Gatti, Adriana Albanese, Diego Nutter, Matías Ezequiel Aguirre, Yésica Dolores Di Iorio, María Celina Elissondo

**Affiliations:** 1 Instituto de Investigaciones en Producción Sanidad y Ambiente (IIPROSAM CONICET-UNMdP), Facultad de Ciencias Exactas y Naturales–UNMdP, Centro Científico Tecnológico Mar del Plata—CONICET, Centro de Asociación Simple CIC PBA, Mar del Plata 7600, Argentina; gisellefuentes07@gmail.com (G.F.); patricia_pensel@hotmail.com (P.E.P.); florgatti24@hotmail.com (F.G.); albanese@mdp.edu.ar (A.A.); 2 Laboratorio de Zoonosis Parasitarias, Facultad de Ciencias Exactas y Naturales (FCEyN), Universidad Nacional de Mar del Plata, Mar del Plata 7600, Argentina; 3 Centro de Investigaciones en Abejas Sociales, Facultad de Ciencias Exactas y Naturales, Universidad Nacional de Mar del Plata, Mar del Plata 7600, Argentina; 4 Departamento de Química y Bioquímica, Facultad de Ciencias Exactas y Naturales, Universidad Nacional de Mar del Plata, Mar del Plata 7600, Argentina; farmramirez@yahoo.com.ar (C.L.R.); meaguirre@mdp.edu.ar (M.E.A.); ydiiorio@mdp.edu.ar (Y.D.D.I.); 5 Asociación Civil CBG2000, Mar del Plata 7600, Argentina; diegonutter1@gmail.com; 6 Instituto de Investigaciones Físicas, Universidad Nacional de Mar del Plata, Mar del Plata 7600, Argentina

**Keywords:** *Echinococcus granulosus sensu stricto*, cystic echinococosis, cannabidiol, phytomedicine

## Abstract

Cystic echinococcosis is a global parasitic zoonosis caused by infection with the larval stage of *Echinococcus granulosus sensu lato*. Cystic echinococcosis affects more than 1 million people worldwide, causing important economic costs in terms of management and livestock associated losses. Albendazole is the main drug used in treating human cystic echinococcosis. In spite of this, its low aqueous solubility, poor absorption, and consequently erratic bioavailability are the cause of its chemotherapeutic failures. Based on the described problem, new treatment alternatives urgently need to be developed. The aim of the present research was to study the *in vitro* and *in vivo* efficacy of cannabidiol (CBD), the second most abundant component of the *Cannabis sativa* plant, was demonstrated against *E. granulosus sensu stricto*. CBD (50 µg/mL) caused a decrease in protoscoleces viability of 80 % after 24 h of treatment which was consistent with the observed tegumental alterations. Detachment of the germinal layer was observed in 50 ± 10% of cysts treated with 50 µg/mL of CBD during 24 h. In the clinical efficacy study, all treatments reduced the weight of cysts recovered from mice compared with the control group. However, this reduction was only significant with ABZ suspension and the CBD + ABZ combination. As we could observe by the SEM study, the co-administration of CBD with ABZ suspension caused greater ultrastructural alteration of the germinal layer in comparison with that provoked with the monotherapy. Further *in vivo* research will be conducted by changing the dose and frequency of CBD and CBD + ABZ treatments and new available CBD delivery systems will also be assayed to improve bioavailability *in vivo*.

## 1. Introduction

The larval stage of *Echinococcus granulosus sensu lato* (*s.l.*) is the cause of cystic echinococcosis, a worldwide parasitic zoonosis. To fulfill its life cycle, the parasite requires the presence of a definitive host (canids, mainly domestic dogs) and an intermediate host (livestock like cattle, goats, camels, swine or sheep). Humans act as dead-end intermediate hosts and can become infected by accidentally ingesting parasite eggs found in the feces of parasitized dogs following different routes of contamination such as ingestion of contaminated food, water or soil, and interaction with contaminated fomites [1]. In humans, the parasite develops into fluid-filled cysts that mainly affect the liver and the lungs, but it can potentially impact any organ or tissue [2]. Within an intermediate host, cysts may also disseminate to other tissues in the case of a cyst rupturing [2].

Cystic echinococcosis affects more than 1 million people worldwide. It causes annual economic costs of more than USD 3 billion in terms of economic losses through management and livestock associated losses such as condemned carcasses and reduced carcass weights, wool, milk and meat, and a substantial public health burden [3]. 

The chosen treatment approach for human is conditioned by diagnostic imaging characteristics, the cyst localization, available surgical/medical expertise and technology, and the possibility of a long-term treatment followed by the patients. For uncomplicated liver and/or abdominal cysts, four principal approaches are applied: (1) treatment with a benzimidazoles; (2) percutaneous aspiration, injection of chemicals, and reaspiration (PAIR); (3) surgery and (4) watch-and-wait [4]. 

Albendazole is the drug used for treatment of human cystic echinococcosis. In spite of this, it has low aqueous solubility, poor absorption, and consequently erratic bioavailability causing its pharmacotherapeutic failures [5]. The ABZ treatment must be administered for a long period of time (4 cycles of 30 days, administered without interruption) [6] leading to undesirable side effects such as hepatotoxicity, alopecia, gastrointestinal distress, vertigo, and leukopenia [5].

Based on the aforementioned facts, there is an urgent need for more effective drugs for the pharmacological treatment of human cystic echinococcosis.

Plant extracts have been used throughout history to treat human and animal diseases due to the fact that they are composed of a vast array of compounds that play specific roles in metabolism. For example, the analgesic properties of *Papaver somnniferum* (poppy) was described 4000 years ago [7]. Plant extracts are the source of natural compounds which can display a significant chemical diversity compared to synthetic compounds and often induce specific biological activities [8]. Medicinal plants have been employed for centuries in the mitigation of parasitic diseases, and these applications persist in numerous regions worldwide [8]. Numerous extracts from plants have exhibited potential for the development of anthelmintic agents: for example, the use of medicinal plant extracts and purified condensed tannins against free-living and parasitic stages of *Oesophagostomum dentatum*; the assay of *Arundo donax*, *Areca catechu*, and *Ferula assafoetida* against *Haemonchus contortus*; and the study of the *in vitro* anthelminthic efficacy of aqueous pomegranate extracts against gastrointestinal nematodes of sheep [8]. In particular, for *E. granulosus* it has been proposed that several plant species and compounds could be potentially used for the development of effective drugs [9].

Plants synthesize a wide range of secondary metabolites including coumarins, flavonoids, tannins, chalcones, terpenoids, and alkaloids, most of which confer the potential anthelmintic activity [10]. The anthelmintic properties of these secondary metabolites can be due to the ability to act as neurotoxin (isoflavonoid-deguelin) [11], their antioxidant properties (carvacrol and thymol) [12], and the uncoupling of mitochondrial oxidative phosphorylation (polygodial) [13]. Furthermore, extensive chemical and functional investigations are necessary to identify the active compounds and comprehend their mechanisms of action [14].

*Cannabis sativa* L., which is an annual herbaceous plant, has been employed for centuries in textiles, food, medicine, and recreational and 1 [15]. Many cultures have well-documented use of *Cannabis* for medicinal purposes. Chinese culture indicates it for rheumatic pain, intestinal constipation, menstrual disorders, and malaria; in the ancient Egypt it was used as an antiinflammatory agent for the eyes; and in India, as a part of the mixture to suppress anxiety [16]. Moreover, it has been utilized in the treatment of a wide variety of other diseases including epilepsy, seizure disorders, depression, insomnia, pain, asthma, nausea, and diarrhea [17]. 

The primary non-psychoactive constituent of the *C. sativa* L., known as cannabidiol (CBD), represents 1 among over 120 cannabinoids that can be isolated from the plant. Many of these cannabinoids have demonstrated biological activity. CBD exhibits notable polypharmacology and has undergone extensive evaluation for diverse disease indications. It possesses anti-inflammatory, neuroprotective, and antimicrobial properties [18]. 

Cannabinoids have the ability to affect various types of infectious agents. When parasites were treated *in vitro* or *in vivo* with cannabinoids, it has been observed to inhibit the proliferation, growth, and invasion of these pathogens [17]. According to the etiologic agent causing the infectious and parasitic diseases, treatments administered together with cannabinoids can significantly interfere with the control of the parasitemia and the clinical outcome of the disease [19].

The aim of our study was to demonstrate the *in vitro* efficacy of CBD against protoscoleces and murine cysts of *E. granulosus sensu stricto* (*s.s*.) The clinical efficacy of CBD in a murine model of cystic echinococcosis was also investigated.

## 2. Material and Methods

### 2.1. Plant Material

Inflorescences were supplied by the NGO CBG 2000 from Mar del Plata city, Buenos Aires, Argentina, and the voucher was issued by the MDQ Herbarium of Vascular Plants of the Plant Diversity Laboratory of the Marine and Coastal Research Institute (IIMyC) of the National University of Mar del Plata (voucher number IMyCHer:MDQ:00630).

### 2.2. Cannabidiol Extraction and Purification

Inflorescences (50 g) were decarboxylated at 120 °C for 40 min and then chopped. The resultant material was suspended in ethanol under vigorous agitation for a period of 30 min. This process was iteratively performed three times on the initial material. The resultant fractions were combined and subjected to desiccation through rotary evaporation. Subsequently, the resulting extract was subjected to purification employing a chromatography column packed with silica gel (Merck 60, 0.015–0.040). The solvent employed in this process comprised hexane and ethyl acetate in varying proportions, transitioning from 90:10 to 30:70. This step was conducted twice to enhance the efficacy of purification outcomes. The resultant product underwent characterization utilizing High-performance Liquid Chromatography (HPLC) [20]. Quantification of cannabidiol (CBD) to ascertain its degree of purity was executed employing a reverse-phase Zorbax SB-Aq column with dimensions of 4.6 mm ID × 250 mm, possessing a particle size of 5 µm. An elution mixture of methanol and ultrapure water (85:15) at a flow rate of 1 mL min^−1^ was utilized, and detection was carried out at λ = 220 nm using a UV–Visible photodiode array detector (UV2000-Thermo Separation Products). The final concentration of CBD was determined through the application of the external standard calibration curve method, resulting in an achieved purity of 99.8%.

### 2.3. Chemicals

Standard solution of cannabidiol (CBD) was purchased from Restek (Bellefonte, PA, USA). Solvents ethanol, methanol and hexane: ethyl acetate were purchased from Sintorgan (Buenos Aires, Argentina), and used as received.

For *in vitro* studies, cannabidiol (CBD) was solubilized in dimethyl sulfoxide (DMSO) at a concentration of 15 mg/mL. Subsequently, it was introduced into the culture medium, yielding final concentrations of 50, 10, 5, and 1 µg/mL. Importantly, the quantity of DMSO introduced into the culture medium did not exceed 3 µL/mL.

For *in vivo* studies, a suspension of albendazole (ABZ) at a concentration of 5.25 mg/mL was formulated using pharmaceutical-grade ABZ (Parafarm, Buenos Aires, Argentina). This formulation involved dispersing pure ABZ in distilled and deionized water (pH = 7.0), subjecting it to overnight shaking, and subsequently, to sonication for 30 min. Concurrently, cannabidiol (CBD) was dissolved in sesame oil (Nutra sem, Buenos Aires, Argentina) at a drug concentration of 4 mg/mL through a one-hour sonication process.

### 2.4. Parasite Material, Protoscoleces Collection and Cyst Obtention

Liver and lung hydatid cysts were procured from cattle slaughtered at an abattoir situated in the Buenos Aires province, Argentina. Protoscoleces were extracted from cysts aseptically and viability was assessed by the methylene blue exclusion test [21].

The parasitic material underwent genotyping through the sequencing of a fragment within the gene responsible for encoding mitochondrial cytochrome c oxidase subunit 1 (CO1), following established protocols [22]. 

To obtain the murine cysts, female CF-1 mice with a body weight of 25 g ± 5 were subjected to intraperitoneal inoculation with 1500 protoscoleces of *E. granulosus s.s.* (G1 genotype) per animal. The protoscoleces were suspended in 0.5 mL of medium 199 (Mediatech, Austin, TX, USA). Six months post-inoculation, mice exhibiting experimental secondary cystic echinococcosis were euthanized, and necropsy was carried out immediately thereafter. During necropsy, the peritoneal cavity was opened, and meticulous removal of the hydatid cysts was performed.

### 2.5. In Vitro Assays

#### 2.5.1. Protoscolicidal Activity 

The culture of 2000 free and viable protoscoleces per Leighton tube was carried out in 6 mL of culture medium 199 at 37 °C without changing the medium during the entire experiment. CBD was added and the final concentrations were 50, 10, 5, and 1 µg/mL. Culture medium with DMSO (3 µL/mL) was used to incubate control protoscoleces. Cultures were conducted in triplicate and the experiment was repeated three times. Microscopy was used every day to observe culture tubes and determine the appearance of morphological alterations. Viability assessment using the methylene blue exclusion test was performed regularly. Ultrastructure studies using scanning electron microscopy (SEM) were conducted periodically on samples of protoscoleces from each of the treatment groups and the control.

#### 2.5.2. Cysticidal Activity

Leighton tubes containing 6 mL of medium 199 were used to place groups of 10 cysts. The medium was supplemented with CBD, leading to final concentrations of 50, 10, 5, and 1 µg/mL. Culture medium with DMSO (3 µL/mL) was used to incubate cysts as a control. Throughout the experiment, the medium in culture tubes was maintained at 37 °C without any changes. Cultures were carried out in triplicate and the experiment was repeated twice. Culture tubes were observed macroscopically and microscopically every day. The criteria for cyst viability assessment included the loss of turgidity and the collapse of the germinal layer [23].

### 2.6. In Vivo Clinical Efficacy Study

Female CF-1 mice (body weight 25 ± 5 g) were infected with 1500 *E. granulosus s.s.* protoscoleces/animal via intraperitoneal injection in medium 199. At 6 months post-infection, female CF-1 mice (*n* = 30) were allocated into the following experimental groups (10 animals/group): (1) Control group, animals treated with water + oil, (2) ABZ group, animals treated with ABZ suspension (25 mg/kg); (3) CBD group, animals treated with CBD (20 mg/kg); (4) ABZ + CBD group, animals treated with ABZ suspension (25 mg/kg) + CBD (20 mg/kg). Treatments were performed by intragastric administration every 24 h for 30 days. All mice were euthanized at the end of the treatment period. The necropsy was performed immediately after and the peritoneal cavity was opened to remove the hydatid cysts with care. The weight of the cysts collected from each animal was registered. Samples of cysts from each group were taken and fixed for histopathological analysis and SEM.

### 2.7. Electron Microscopy

Samples of protoscoleces taken from the *in vitro* studies and samples of metacestodes taken from *in vivo* studies were fixed with 3 % glutaraldehyde in sodium cacodylate buffer for 72 h at 4 °C and then washed three times with cacodylate buffer. In order to dehydrate the parasites, they were incubated sequentially in increasing concentrations of ethanol (50 to 100%) and hexamethyldisilazane. Afterwards, the samples were coated with gold (100-Å thickness) and examined using a JEOL JSM-6460 LV scanning electron microscope operated at 15 kV.

### 2.8. Statistical Analysis

Both statistical analyses and final figures were carried out in the R program (version 4.3.1) [24]. All tests were considered statistically significant when P values were less than 0.05. For evaluating the effect of CBD on protoscoleces and cysts of *E. granulosus s.s.*, a generalized linear model was applied. Moreover, the differences between pairs of conditions (Control, DMSO, and CBD concentrations were tested using the ‘emmeans’ package [25]. To measure differences in cyst weight between treated groups in the *in vivo* experiments, the Kruskal-Wallis test and Dunn’s multiple comparisons test were employed.

### 2.9. Ethic Statement and Experimental Animals

Protocols for the animal procedures and management were approved by the Institutional Animal Care and Use Committee (RD No. 40/2022) of the Faculty of Exact and Natural Sciences, National University of Mar del Plata, Argentina and performed in accordance with the revised form of The Guide for the Care and Use of Laboratory Animals [26]. The study prevented unnecessary animal suffering throughout. Animals were confined to a room that was temperature-controlled and had a 12 h light/dark cycle with a temperature of 22 ± 1 °C. Ad libitum food and water were available.

## 3. Results

**Figure 1:**
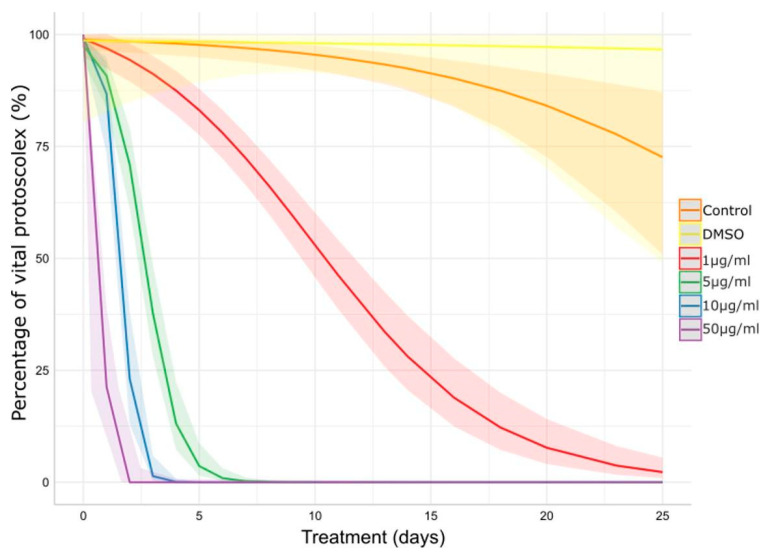
Impact of CBD on *E. granulosus s.s.* protoscoleces viability assessed by methylene blue exclusion test. The lines and ribbons show the predicted fits and 95% confidence intervals. DMSO: dimethyl sulfoxide.

The *in vitro* protoscolicidal effect of CBD is illustrated in Figure 1. The scolicidal activity of CBD increased dose-time dependently (*p* < 0.001). The control group remained vital throughout the experimental period and no morphological changes were observed. The concentration of 50 μg/mL of CBD resulted in a 50% reduction in vitality before the first 24 h of treatment and 0% of vitality at 48 h post-treatment. Concentrations of 10 and 5 μg/mL also caused a rapid viability decrease, reaching 0% between days 3–4 and 6–7, respectively. The concentration of 1 μg/mL caused a gradual decrease in vitality, reaching values of 50% on day 11, approximately, and 5% at the end of the experiment.

**Figure 2:**
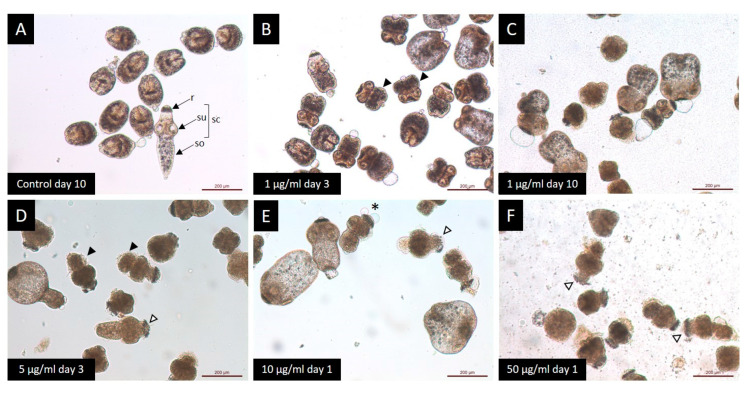
Light microscopy of *E. granulosus s.s.* protoscoleces treated *in vitro* with different CBD concentrations. (**A**) Observe the typical protoscoleces morphology: (r) rostellum, (su) suckers, (so) soma and (sc) scolex. Note the presence of morphological alterations: (**B**,**D**) soma contraction (black arrow head); (**C**) tegumental damage; (**E**) presence of blebs (*); and (**E**,**F**) rostellar disorganization (white arrow head).

**Figure 3:**
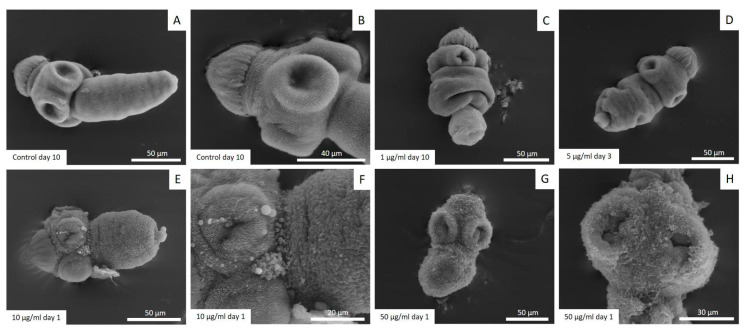
Representative images from scanning electron microscopy of *E. granulosus s.s.* protoscoleces treated *in vitro* with different CBD concentrations. Note the normal ultrastructure of protoscoleces (**A**,**B**), soma contraction (**C**), tegumental damage (**D**), presence of blebs in the tegument (**E**,**F**) and severe tegumental damage (**G**,**H**).

The results of the protoscoleces vitality test were consistent with the observed structural (Figure 2) and ultrastructural (Figure 3) damages. Control protoscoleces maintained their normal morphology throughout the experiment consisting of soma and scolex with suckers (Figure 2A) covered with microtriches (Figure 3A,B). The treatment with 1 μg/mL of CBD for 3 days caused soma contraction (Figure 2B and Figure 3C) and after 10 days several protoscoleces with tegumental damage could be observed (Figure 2C). After 3 days of incubation with 5 μg/mL of CBD, protoscoleces showed soma contraction and hook loss (Figure 2D), and severe damage in the tegument (Figure 3D). Protoscoleces treated with 10 μg/mL of CBD for 1 day showed rostellar disorganization (Figure 2E) and the presence of blebs in the tegument (Figure 3E,F).

The treatment with the highest concentration (50 μg/mL) for 1 day caused severe damage such as rostellar disorganization and rostellum loss (Figure 2F) and profound tegumental alterations (Figure 3G,H).

**Figure 4:**
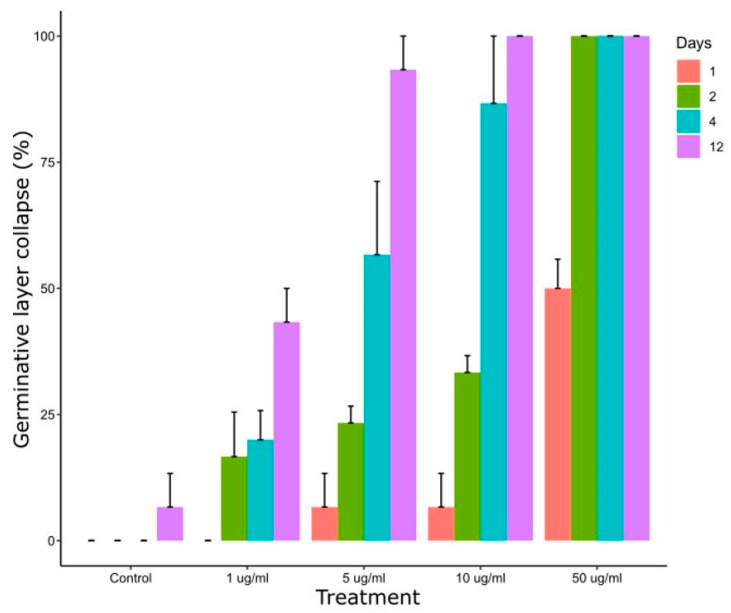
Collapse of the germinal layer of *E. granulosus s.s.* cysts after 1, 2, 4 and 12 days of *in vitro* exposure to different CBD concentrations.

Figure 4 shows the effect of CBD on *E. granulosus s.s.* cysts after *in vitro* exposure to different concentrations. After 4 days of treatment, collapse of the germinal layer was observed in 20 ± 10%, 57 ± 25% and 87 ± 23% of cyst incubated with 1 μg/mL, 5 μg/mL and 10 μg/mL, respectively. Treatment with 50 µL/mL of CBD for 24 h and 48 h caused a collapse of the germinal layer of 50 ± 10% and 100% of cysts, respectively.

**Figure 5:**
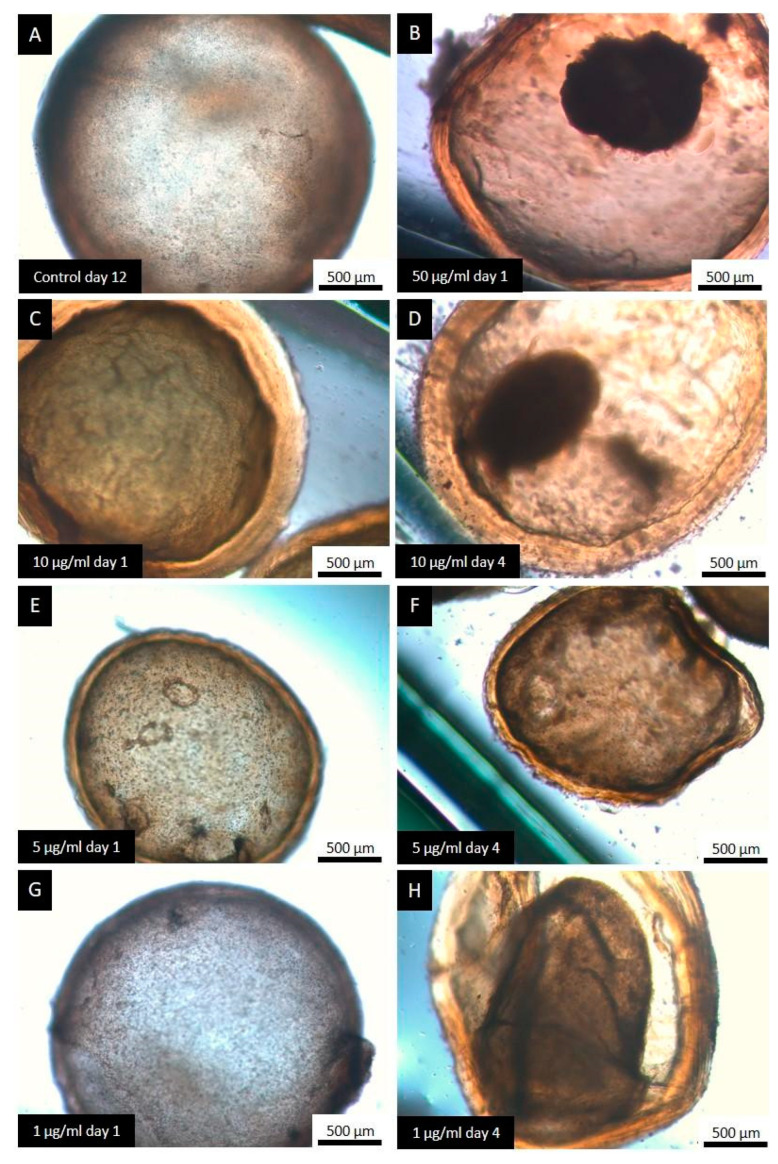
Representative images from the light microscopy of *E. granulosus s.s.* cysts treated with the different concentrations of CBD. Observe the normal structure of a cyst formed by an inner cellular layer (germinal layer) and an external acellular laminated layer (**A**), the different alterations caused by the treatment with CBD: unaltered cysts (**E**,**G**), loss of turgency (**C**), complete detachment of the germinal layer (**B**,**D**), and initiation of germinal layer detachment (**F**,**H**).

Control cysts maintained their normal structure and were macroscopically turgid throughout the *in vitro* incubation period (Figure 5A). All concentrations tested caused alterations from the first days post-incubation, such as loss of turgidity (Figure 5C), initiation of germinal layer collapse (Figure 5F,H), and complete collapse of the germinal layer (Figure 5B,D). The occurrence of collapsed cysts was proportional to the concentration used.

The parasitic material used in all the experiments was identified as G1 genotype by the sequencing analysis.

The animals exhibited normal behavior and appearance throughout the entire clinical efficacy study. Table 1 provides a summary of the cyst weights (median and IQR) collected after treatments for the different experimental groups involved in the study. The weight of cysts recovered from treated mice was lower compared to the control group. However, this reduction was only significant with ABZ suspension and the CBD + ABZ combination. 

**Figure 6:**
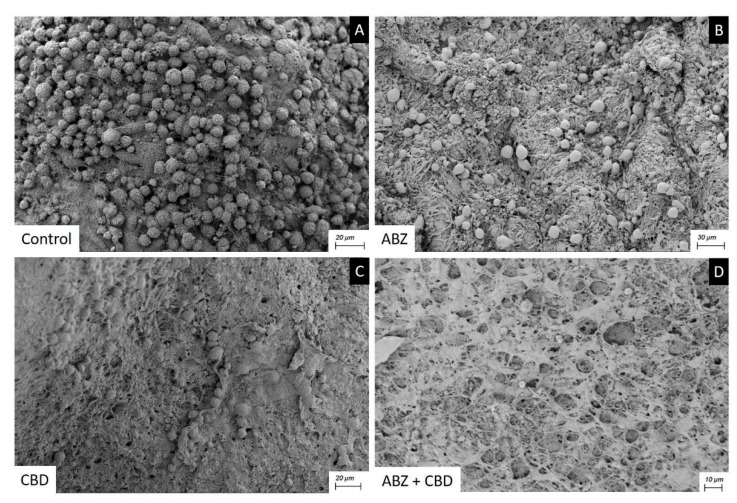
Scanning electron microscopy of *E. granulosus s.s.* cysts recovered after clinical efficacy study. Note the normal ultrastructure of the germinal layer (**A**), the effect of CBD and ABZ causing a reduction in cell number and damaged cells (**B**,**C**), the greater impact of ABZ+CBD on the germinal layer (**D**).

Cysts recovered from the control group studied by SEM showed an intact germinal layer formed by a multitude of different cell types (Figure 6A).

After receiving ABZ 25 mg kg^−1^ (Figure 6B) or CBD 20 mg kg^−1^ (Figure 6C) therapy, cysts recovered showed a germinal layer with a decrease in cell numbers and damaged cells. The ultrastructural changes seen in the cysts recovered from mice treated with ABZ + CBD were much greater than those caused by monotherapy (Figure 6D).

## 4. Discussion

In search of new, safer, and more effective therapeutic options, we showed the *in vitro* efficacy of CBD against protoscoleces and murine cysts of *E. granulosus s.s*., and the clinical efficacy of CBD in a murine model of cystic echinococcosis was also demonstrated.

Several studies have been carried out in recent years using different medicinal plants on *E. granulosus s.l.* However, research on the isolation and purification of plant compounds with antiparasitic properties is notably limited and inadequate. On the other hand, for evaluation of scolicidal activity, mostly essential oils were utilized [8]. Among the few isolated compounds reported are thymol (main phenolic component of thyme essential oil), carvacrol (major component of oregano essential oil), cinnamaldehyde (the main component of *Cinnamomum zeylanicum* essential oil), beta-myrcene (an acyclic monoterpene main component of the *Rosmarinus officinalis* essential oil), berberine (an alkaloid widely used in traditional Chinese medicines and ayurveda), and thymoquinone (the main component of essential oil of *Nigella sativa*). They revealed significant *in vitro* scolicidal activity [23,27,28,29,30,31] although the *in vivo* effect was only assayed for thymol, carvacrol and beta-myrcene. After oral administration of 40 mg/kg of thymol both chemoprophylactic and clinical efficacy studies demonstrated it to be effective [32]. The administration of 40 mg/kg of carvacrol caused a reduction in cyst weight compared to control mice [23].

In both cases the preventive effect was comparable with the effect of ABZ suspension. In contrast, beta-myrcene caused a low *in vivo* efficacy on cysts growth [30].

*Cannabis sativa* was used for thousands of years for several ailments, but its psychoactive effects and recreational use caused it to be considered a forbidden substance for a long time [33]. Scientific interest in Cannabis has increased considerably since the discovery in 1990s of the endocannabinoid system (endogenous ligands, cannabinoid receptors, and enzymatic machinery) and the acknowledgment of its role in health and disease [16].

As one of the main active components of the *C. sativa* extract and due to the lack of psychoactivity and its safety profile, CBD is undoubtedly the more intriguing cannabinoid with numerous reported pharmacological effects. It is used for the treatment of different pathologies, such as inflammatory and neurodegenerative diseases, epilepsy, multiple sclerosis, arthritis, schizophrenia and cancer [34]. Moreover, very recently the potential use of CBD against parasitic infection has been highlighted [35]. It has been reported that treatment with CBD (30 mg/kg for 7 days) in a murine model of cerebral infection with *Plasmodium berghei,* caused lower parasite load in the brain and prevented memory deficits and anxiety behavior leading to a higher survival rate [19]. The treatment of *Riphicephalus microplus*, an important livestock tick, with 40 mg/mL of a *C. sativa* extract caused a significant negative impact on egg hatching, egg laying and larval mortality [36].

The treatment with CBD caused a marked scolicidal effect and it was in accordance with the alterations observed with the different CBD concentrations such as soma contraction, loss of hooks, presence of blebs in the tegument and severe tegumetal alterations. On the other hand, a rapid germinal layer detachment could be observed with the concentration of 50 µL/mL reaching 100% of cysts collapsed after 48 h post-incubation. Ultrastructural alterations observed in protoscoleces treated *in vitro* with CBD were similar to those caused by other natural products such as beta-myrcene, carvacrol and thymol [23,30,37].

All treatments reduced the weight of cysts recovered from mice compared with the control group during the clinical efficacy study. However, this reduction was only significant with ABZ suspension and the CBD + ABZ combination. As we could observe by the SEM study, the co-administration of CBD with ABZ suspension caused greater ultrastructural alteration of the germinal layer in comparison with that provoked with the monotherapy.

Similarly to the echinococcosis reference drug ABZ, CBD has a low bioavailability, very low water solubility and high lipophilicity [38]. Different cannabidiol delivery systems that have been developed, and applied therapeutically have been recently summarized [39]. Some of these systems include nanolipospheres, ethosomes, microparticles, nanocrystals, and nanoparticles and it has been proposed that they are able to improve the dissolution profile of CBD by protecting it from metabolization and producing the release in a specific site that increases its bioavailability, making CBD administration clinically effective.

Based on the promising results obtained in our study, it would be interesting to assay in the future some of the aforementioned available CBD delivery systems to improve bioavailability *in vivo*, and thus enhance the efficacy on *E. granulosus s.s.*

The CBD dose employed in this study (20 mg kg^−1^) was previously used in other murine models [40,41] and also it is considered a safe dose as reported by Ewing et al. [42] where deleterious effects were found at much higher doses. They demonstrated that a concentrated CBD-enriched cannabis extract delivered orally to mice, has the potential to cause hepatotoxicity at very high doses (2460 mg/kg). Nonetheless, mice gavaged with CBD at 184.5 mg/kg or lower did not display any toxicological responses associated with liver injury.

Concerning the proposed mechanism of action for CBD, multiple studies point to the alteration of membrane permeability. CBD was effective on *Staphylococcus aureus*, causing the depolarization of the cytoplasmic membrane and the disruption of the membrane potential. Another putative mode of action of CBD is the inhibition of membrane vesicles releasing causing alterations on cell communication [16].

The outer surfaces of helminths function as a barrier that shields the organism from external conditions. The tegumental syncytium in flatworms performs vital functions for nutrient uptake, immunoprotection, osmoregulation, and structural support [43]. Lipophilic anthelmintic molecules accumulate in target parasites due to passive drug transfer through the external helminth surface [44]. CBD as a lipophilic drug acting on the membrane permeability together with the structure of the external surface of the parasite could be the factors that affect the entry of the drug into the protoscoleces and cysts to produce the anthelmintic effect.

In this study we reported for the first time the *in vitro* effect of CBD on *E. granulosus s.s.* protoscoleces and cyst and also the *in vivo* efficacy on a murine model of CE. Further *in vivo* research will be conducted by changing the dose and frequency of CBD and CBD + ABZ treatments and also new available CBD delivery systems will be assayed to improve the bioavailability *in vivo*.

## Figures and Tables

**Table 1:** Clinical efficacy study. Interquartile range (IQR) and median weight (g) of the *E. granulosus s.s.* cyst obtained from experimentally infected mice from the control and treated groups.

Group	Median Weight of Cysts (g)	Interquartile Range (IQR)
Control	1.55	2.5
ABZ	0.1 *	0.77
CBD	0.83	1.54
ABZ + CBD	0.24 *	0.25

* Statistically significant differences with the control group (*p* < 0.05).

## Data Availability

The raw data supporting the conclusions of this article will be made available by the authors on request.
